# 2-[2-(Benzyl­sulfan­yl)phen­yl]-1,1,3,3-tetra­methyl­guanidine

**DOI:** 10.1107/S1600536811014577

**Published:** 2011-04-22

**Authors:** Adam Neuba, Ulrich Flörke, Gerald Henkel

**Affiliations:** a Department Chemie, Fakultät für Naturwissenschaften, Universität Paderborn, Warburgerstrasse 100, D-33098 Paderborn, Germany

## Abstract

The mol­ecular structure of the title compound, C_18_H_23_N_3_S, shows it to be a derivative of an amino­thio­phenol possessing a tetra­methyl­guanidine group with a localized C=N double bond of 1.304 (2) Å and a protected thiol functional group as an *S*-benzyl thio­ether. The two aromatic ring planes make a dihedral angle of 67.69 (6)°.

## Related literature

For synthesis, see: Neuba (2009 ▶); Lindoy & Livingstone (1968 ▶); Herres-Pawlis *et al.* (2005 ▶). For related structures, see: Neuba *et al.* (2007*a* ▶,*b* ▶,*c* ▶); Herres *et al.* (2004 ▶); Raab *et al.* (2003 ▶, 2002 ▶); Peters *et al.* (2008 ▶). For complexes of metal centres with bis­(tetra­methylguanidino)propyl­ene and amine guanidine hybrids, see: Harmjanz (1997 ▶); Waden (1999 ▶); Pohl *et al.* (2000 ▶); Schneider (2000 ▶); Wittmann (1999 ▶); Wittmann *et al.* (2001 ▶); Herres *et al.* (2005 ▶); Herres-Pawlis *et al.* (2009 ▶); Börner *et al.* (2007 ▶, 2009 ▶). For sulfur guanidine hybrids based on amino­thio­phenol and cysteamine, see: Neuba (2009 ▶); Neuba *et al.* (2008*a* ▶,*b* ▶, 2010 ▶, 2011 ▶).
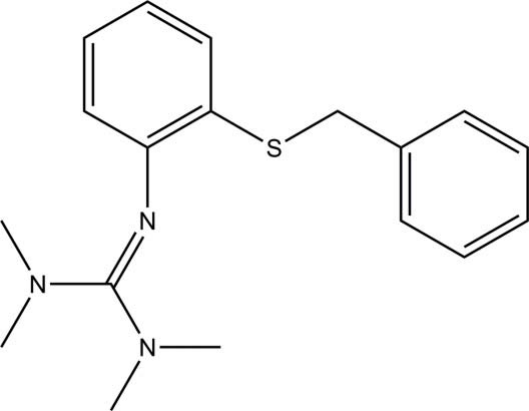


## Experimental

### 

#### Crystal data

C_18_H_23_N_3_S
*M* *_r_* = 313.45
Monoclinic, 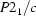

*a* = 7.869 (2) Å
*b* = 26.850 (7) Å
*c* = 8.314 (2) Å
β = 106.959 (5)°
*V* = 1680.2 (8) Å^3^
*Z* = 4
Mo *K*α radiation
μ = 0.19 mm^−1^
*T* = 120 K
0.42 × 0.33 × 0.29 mm

#### Data collection

Bruker SMART APEX diffractometer
Absorption correction: multi-scan (*SADABS*; Sheldrick, 2004 ▶) *T*_min_ = 0.923, *T*_max_ = 0.946
14278 measured reflections
3988 independent reflections
2816 reflections with *I* > 2σ(*I*)
*R*_int_ = 0.066

#### Refinement

*R*[*F* ^2^ > 2σ(*F* ^2^)] = 0.041
*wR*(*F* ^2^) = 0.091
*S* = 0.92
3988 reflections
203 parameters
H-atom parameters constrained
Δρ_max_ = 0.24 e Å^−3^
Δρ_min_ = −0.34 e Å^−3^

### 

Data collection: *SMART* (Bruker, 2002 ▶); cell refinement: *SAINT* (Bruker, 2002 ▶); data reduction: *SAINT*; program(s) used to solve structure: *SHELXTL* (Sheldrick, 2008 ▶); program(s) used to refine structure: *SHELXTL*; molecular graphics: *SHELXTL*; software used to prepare material for publication: *SHELXTL* and local programs.

## Supplementary Material

Crystal structure: contains datablocks I, global. DOI: 10.1107/S1600536811014577/bt5518sup1.cif

Structure factors: contains datablocks I. DOI: 10.1107/S1600536811014577/bt5518Isup2.hkl

Additional supplementary materials:  crystallographic information; 3D view; checkCIF report

## Figures and Tables

**Fig. 1.**
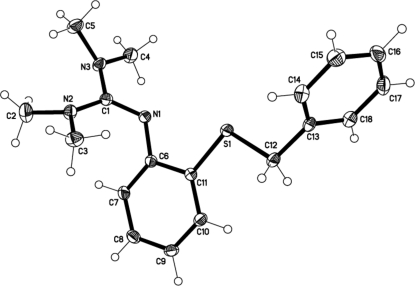
Molecular structure with displacement ellipsoids drawn at the 50% probability level.

## supplementary crystallographic information

### Comment

The synthesis and characterization of novel molecules containing nitrogen and sulfur as donor functions and their application in synthesis of sulfur copper complexes is important for biomimetic copper–sulfur chemistry. In search of multifunctional ligands we have extended our studies to guanidyl-type systems with N-donor functions. The first derivative, the ligand bis(tetramethyl-guanidino)propylene as well as amine guanidine hybrids and their complexes with Cu, Fe, Ni, Ag, Mn, Co and Zn have recently been investigated (Harmjanz, 1997; Waden, 1999; Pohl *et al.*, 2000; Schneider, 2000; Wittmann, 1999; Wittmann *et al.*, 2001; Herres-Pawlis *et al.*, 2005, 2009; Herres *et al.*, 2005; Neuba *et al.*, 2008*a*,*b*; 2010; Börner *et al.* 2007, 2009). We have now developed several sulfur guanidine hybrids based on aminothiophenol and cysteamine (Neuba *et al.*, 2007*a*,*b*,*c*; Neuba, 2009). The synthesized sulfur guanidine compounds possess aliphatic and aromatic thioethers or disulfide groups and were used in the synthesis of copper thiolate complexes to mimic active centres like the Cu_A_ in cytochrome-c oxidase and N_2_O-reductase (Neuba *et al.*, 2011). The two aromatic ring planes make a dihedral angle of 67.69 (6)° and the N1—C6—C11—S1—C12—C13 moiety is mostly planar with largest deviation of 0.084 (1) Å for S1 from the best plane. The guanidine plane C1N_3_ makes an angle of 59.80 (5)° with the attached aromatic ring. The N1═C1 guanidine double bond measures 1.304 (2) Å and is clearly localized. Similar double-bond localization is observed in other guanidine compounds (*e.g.* Herres *et al.*, 2004; Neuba *et al.*, 2007*a*,*b*; Raab *et al.*, 2002, 2003; Peters *et al.*, 2008).

### Experimental

The title compound was prepared as follows: a solution of tetramethylchloroformamidinium chlorid (Herres-Pawlis *et al.*, 2005) (5.13 g, 30 mmol) in dry MeCN was added dropwise to an ice-cooled solution of 2-(benzylthio)aniline (Lindoy & Livingstone, 1968) (6.45 g, 30 mmol) and triethylamine (4.18 ml, 3.03 g, 30 mmol) in dry MeCN. After 3 h under reflux, a solution of NaOH (1.2 g, 30 mmol) in water was added. The solvents and NEt_3_ were then evaporated under vacuum. In order to deprotonate the mono-hydrochloride, 50 wt% KOH (aqueous, 15 ml) was added and the free base was extracted into the MeCN phase (3 × 80 ml). The organic phase was dried with Na_2_SO_4_. After filtration, the solvent was evaporated under reduced pressure. The title compound was obtained as white powder (yield 69%, 6.4 g). Colourless crystals suitable for X-ray diffraction were obtained by slow cooling of a hot saturated MeCN solution.

Spectroscopic analysis: ^1^H NMR (500 MHz, CDCl_3_, 25°C, δ, p.p.m.): 2.68 (s, 12H, CH_3_), 4.10 (s, 2H, CH_2_), 6.59 (d, 1H, CH), 6.80 (t, 1H, CH), 7.03 (t, 1H, CH), 7.15 (d, 1H, CH), 7.21 (t, 1H, CH), 7.28 (t, 2H, CH), 7.36 (d, 2H, CH); ^13^C NMR (125 MHz, CDCl_3_, 25°C, δ, p.p.m.): 37.3 (CH_2_), 39.5 (CH_3_), 120.5 (CH), 121.7 (CH), 126.1 (CH), 126.9 (CH), 127.2 (CH), 127.6 (CH), 128.4 (CH), 128.7 (C_quat_), 129.1 (CH), 136.6 (C_quat_), 137.8 (C_quat_), 160.0 (C_gua_); IR (KBr, ν, cm^-1^): 3053 (w), 3030 (w), 3003 (w), 2918 (*m*), 2848 (*m*), 2790 (w), 1589 (*vs* (C═N)), 1558 (s (C═N)), 1500 (m (C═N)), 1460 (*m*), 1425 (*m*), 1377 (*s*), 1279 (w), 1232 (w), 1207 (w), 1144 (*m*), 1066 (*m*), 1038 (*m*), 1020 (*s*), 914 (w), 850 (w), 806 (vw), 777 (*m*), 715 (*s*), 696 (*m*), 682 (w), 621 (w), 571 (w), 545 (w), 498 (vw), 484 (w), 461 (w), 445 (w). EI–MS (*m*/*z* (%)): 313.0 (100) [*M*^+^], 280.0 (31), 269.1 (8) [*M*^+^ - N(CH_3_)_2_], 242.0 (5), 237.0 (10) [*M*^+^ - Ph], 222.0 (14) [*M*^+^ - CH_2_Ph], 215.0 (20), 190.0 (12) [*M*^+^ - SCH_2_Ph], 179.0 (76), 148.9 (28), 135.9 (20), 124.0 (9) [SCH_2_Ph^+^], 91.0 (76), 72.0 (20).

### Refinement

H atoms were clearly identified in difference syntheses, idealized and refined riding on the C atoms with C—H = 0.95 (aromatic) or 0.98–0.99 Å, and with isotropic displacement parameters *U*_iso_(H) = 1.2*U*_eq_(C) or 1.5*U*_eq_(–CH_3_ H atoms). All CH_3_ H atoms were allowed to rotate but not to tip.

### Crystal data

**Table:**

C_18_H_23_N_3_S	*F*(000) = 672
*M**_r_* = 313.45	*D*_x_ = 1.239 Mg m^−^^3^
Monoclinic, *P*2_1_/*c*	Mo *K*α radiation, λ = 0.71073 Å
Hall symbol: -P 2ybc	Cell parameters from 842 reflections
*a* = 7.869 (2) Å	θ = 2.7–27.7°
*b* = 26.850 (7) Å	µ = 0.19 mm^−^^1^
*c* = 8.314 (2) Å	*T* = 120 K
β = 106.959 (5)°	Block, colourless
*V* = 1680.2 (8) Å^3^	0.42 × 0.33 × 0.29 mm
*Z* = 4	

### Data collection

**Table:**

Bruker SMART APEX diffractometer	3988 independent reflections
Radiation source: sealed tube	2816 reflections with *I* > 2σ(*I*)
graphite	*R*_int_ = 0.066
φ and ω scans	θ_max_ = 27.9°, θ_min_ = 1.5°
Absorption correction: multi-scan (*SADABS*; Sheldrick, 2004)	*h* = −10→9
*T*_min_ = 0.923, *T*_max_ = 0.946	*k* = −35→35
14278 measured reflections	*l* = −10→10

### Refinement

**Table:**

Refinement on *F*^2^	Primary atom site location: structure-invariant direct methods
Least-squares matrix: full	Secondary atom site location: difference Fourier map
*R*[*F*^2^ > 2σ(*F*^2^)] = 0.041	Hydrogen site location: difference Fourier map
*wR*(*F*^2^) = 0.091	H-atom parameters constrained
*S* = 0.92	*w* = 1/[σ^2^(*F*_o_^2^) + (0.0443*P*)^2^] where *P* = (*F*_o_^2^ + 2*F*_c_^2^)/3
3988 reflections	(Δ/σ)_max_ = 0.001
203 parameters	Δρ_max_ = 0.24 e Å^−^^3^
0 restraints	Δρ_min_ = −0.34 e Å^−^^3^

### Special details

**Table:**

Geometry. All e.s.d.'s (except the e.s.d. in the dihedral angle between two l.s. planes) are estimated using the full covariance matrix. The cell e.s.d.'s are taken into account individually in the estimation of e.s.d.'s in distances, angles and torsion angles; correlations between e.s.d.'s in cell parameters are only used when they are defined by crystal symmetry. An approximate (isotropic) treatment of cell e.s.d.'s is used for estimating e.s.d.'s involving l.s. planes.
Refinement. Refinement of *F*^2^ against ALL reflections. The weighted *R*-factor *wR* and goodness of fit *S* are based on *F*^2^, conventional *R*-factors *R* are based on *F*, with *F* set to zero for negative *F*^2^. The threshold expression of *F*^2^ > σ(*F*^2^) is used only for calculating *R*-factors(gt) *etc*. and is not relevant to the choice of reflections for refinement. *R*-factors based on *F*^2^ are statistically about twice as large as those based on *F*, and *R*-factors based on ALL data will be even larger.

### Fractional atomic coordinates and isotropic or equivalent isotropic displacement parameters (Å^2^)

**Table:**

	*x*	*y*	*z*	*U*_iso_*/*U*_eq_	
S1	0.48274 (6)	0.392868 (13)	0.76377 (5)	0.02350 (11)	
N1	0.64141 (18)	0.33140 (4)	0.56770 (16)	0.0214 (3)	
N2	0.93294 (17)	0.29594 (4)	0.63993 (16)	0.0232 (3)	
N3	0.68294 (18)	0.24922 (4)	0.50838 (17)	0.0239 (3)	
C1	0.7516 (2)	0.29479 (5)	0.57258 (18)	0.0203 (3)	
C2	1.0512 (2)	0.27118 (7)	0.5595 (2)	0.0362 (4)	
H2A	0.9806	0.2524	0.4615	0.054*	
H2B	1.1294	0.2483	0.6395	0.054*	
H2C	1.1231	0.2961	0.5232	0.054*	
C3	1.0191 (2)	0.33296 (6)	0.7646 (2)	0.0291 (4)	
H3A	1.0587	0.3609	0.7089	0.044*	
H3B	1.1220	0.3179	0.8466	0.044*	
H3C	0.9349	0.3450	0.8225	0.044*	
C4	0.4983 (2)	0.24676 (6)	0.4081 (2)	0.0311 (4)	
H4A	0.4223	0.2445	0.4825	0.047*	
H4B	0.4800	0.2173	0.3354	0.047*	
H4C	0.4678	0.2768	0.3385	0.047*	
C5	0.7491 (2)	0.20329 (5)	0.5999 (2)	0.0309 (4)	
H5A	0.8732	0.2079	0.6666	0.046*	
H5B	0.7414	0.1760	0.5196	0.046*	
H5C	0.6772	0.1952	0.6746	0.046*	
C6	0.6947 (2)	0.38116 (5)	0.56372 (19)	0.0192 (3)	
C7	0.7988 (2)	0.39792 (5)	0.4653 (2)	0.0233 (3)	
H7A	0.8470	0.3744	0.4052	0.028*	
C8	0.8338 (2)	0.44823 (6)	0.4527 (2)	0.0252 (4)	
H8A	0.9042	0.4589	0.3838	0.030*	
C9	0.7656 (2)	0.48271 (5)	0.5412 (2)	0.0236 (3)	
H9A	0.7905	0.5171	0.5343	0.028*	
C10	0.6610 (2)	0.46705 (5)	0.63978 (19)	0.0220 (3)	
H10A	0.6153	0.4908	0.7009	0.026*	
C11	0.6223 (2)	0.41684 (5)	0.65010 (18)	0.0192 (3)	
C12	0.4411 (2)	0.44748 (5)	0.8758 (2)	0.0243 (4)	
H12A	0.5549	0.4615	0.9463	0.029*	
H12B	0.3797	0.4733	0.7947	0.029*	
C13	0.3272 (2)	0.43232 (5)	0.98479 (19)	0.0210 (3)	
C14	0.4001 (2)	0.40508 (6)	1.1318 (2)	0.0274 (4)	
H14A	0.5226	0.3966	1.1635	0.033*	
C15	0.2967 (3)	0.39029 (6)	1.2317 (2)	0.0317 (4)	
H15A	0.3480	0.3719	1.3316	0.038*	
C16	0.1184 (3)	0.40237 (6)	1.1860 (2)	0.0332 (4)	
H16A	0.0467	0.3919	1.2539	0.040*	
C17	0.0442 (2)	0.42969 (6)	1.0417 (2)	0.0329 (4)	
H17A	−0.0781	0.4384	1.0113	0.039*	
C18	0.1484 (2)	0.44444 (6)	0.9414 (2)	0.0266 (4)	
H18A	0.0967	0.4630	0.8420	0.032*	

### Atomic displacement parameters (Å^2^)

**Table:**

	*U*^11^	*U*^22^	*U*^33^	*U*^12^	*U*^13^	*U*^23^
S1	0.0297 (2)	0.01664 (17)	0.0296 (2)	−0.00079 (16)	0.01715 (18)	−0.00202 (15)
N1	0.0224 (7)	0.0167 (6)	0.0277 (7)	−0.0022 (5)	0.0116 (6)	−0.0044 (5)
N2	0.0206 (7)	0.0215 (6)	0.0263 (7)	−0.0002 (5)	0.0047 (6)	−0.0055 (5)
N3	0.0234 (7)	0.0164 (6)	0.0296 (7)	−0.0001 (5)	0.0043 (6)	−0.0045 (5)
C1	0.0237 (9)	0.0187 (7)	0.0199 (8)	−0.0011 (6)	0.0089 (7)	−0.0020 (6)
C2	0.0267 (10)	0.0419 (10)	0.0414 (11)	0.0044 (8)	0.0121 (9)	−0.0069 (8)
C3	0.0288 (10)	0.0255 (8)	0.0281 (9)	−0.0054 (7)	0.0008 (8)	−0.0025 (7)
C4	0.0253 (10)	0.0251 (8)	0.0400 (10)	−0.0031 (7)	0.0049 (8)	−0.0076 (7)
C5	0.0350 (10)	0.0193 (7)	0.0367 (10)	0.0001 (7)	0.0076 (9)	−0.0016 (7)
C6	0.0175 (8)	0.0181 (7)	0.0219 (8)	−0.0009 (6)	0.0058 (7)	−0.0018 (6)
C7	0.0221 (9)	0.0230 (8)	0.0277 (8)	0.0002 (6)	0.0119 (7)	−0.0037 (6)
C8	0.0228 (9)	0.0273 (8)	0.0279 (9)	−0.0026 (7)	0.0111 (7)	0.0035 (7)
C9	0.0242 (9)	0.0168 (7)	0.0289 (9)	−0.0020 (6)	0.0061 (7)	0.0020 (6)
C10	0.0228 (9)	0.0173 (7)	0.0252 (8)	0.0013 (6)	0.0059 (7)	−0.0012 (6)
C11	0.0182 (8)	0.0195 (7)	0.0209 (8)	−0.0004 (6)	0.0071 (7)	−0.0002 (6)
C12	0.0305 (10)	0.0174 (7)	0.0280 (9)	0.0022 (6)	0.0131 (8)	−0.0036 (6)
C13	0.0258 (9)	0.0169 (7)	0.0218 (8)	0.0007 (6)	0.0092 (7)	−0.0061 (6)
C14	0.0259 (9)	0.0296 (8)	0.0267 (9)	0.0056 (7)	0.0076 (8)	−0.0013 (7)
C15	0.0425 (11)	0.0296 (8)	0.0243 (9)	0.0015 (8)	0.0117 (8)	0.0015 (7)
C16	0.0423 (12)	0.0292 (9)	0.0372 (10)	−0.0078 (8)	0.0260 (9)	−0.0093 (7)
C17	0.0224 (10)	0.0332 (9)	0.0451 (11)	0.0010 (7)	0.0129 (9)	−0.0113 (8)
C18	0.0282 (10)	0.0235 (8)	0.0281 (9)	0.0028 (7)	0.0082 (8)	−0.0025 (6)

### Geometric parameters (Å, °)

**Table:**

S1—C11	1.7661 (15)	C6—C11	1.413 (2)
S1—C12	1.8175 (15)	C7—C8	1.389 (2)
N1—C1	1.3035 (19)	C7—H7A	0.9500
N1—C6	1.4033 (18)	C8—C9	1.384 (2)
N2—C1	1.373 (2)	C8—H8A	0.9500
N2—C3	1.4536 (19)	C9—C10	1.386 (2)
N2—C2	1.455 (2)	C9—H9A	0.9500
N3—C1	1.3804 (18)	C10—C11	1.390 (2)
N3—C4	1.451 (2)	C10—H10A	0.9500
N3—C5	1.4628 (19)	C12—C13	1.505 (2)
C2—H2A	0.9800	C12—H12A	0.9900
C2—H2B	0.9800	C12—H12B	0.9900
C2—H2C	0.9800	C13—C18	1.385 (2)
C3—H3A	0.9800	C13—C14	1.396 (2)
C3—H3B	0.9800	C14—C15	1.380 (2)
C3—H3C	0.9800	C14—H14A	0.9500
C4—H4A	0.9800	C15—C16	1.381 (3)
C4—H4B	0.9800	C15—H15A	0.9500
C4—H4C	0.9800	C16—C17	1.382 (2)
C5—H5A	0.9800	C16—H16A	0.9500
C5—H5B	0.9800	C17—C18	1.387 (2)
C5—H5C	0.9800	C17—H17A	0.9500
C6—C7	1.391 (2)	C18—H18A	0.9500
			
C11—S1—C12	102.33 (7)	C8—C7—H7A	119.2
C1—N1—C6	121.20 (13)	C6—C7—H7A	119.2
C1—N2—C3	121.30 (13)	C9—C8—C7	119.62 (14)
C1—N2—C2	122.01 (13)	C9—C8—H8A	120.2
C3—N2—C2	114.35 (14)	C7—C8—H8A	120.2
C1—N3—C4	118.43 (13)	C8—C9—C10	120.05 (14)
C1—N3—C5	120.43 (14)	C8—C9—H9A	120.0
C4—N3—C5	113.92 (13)	C10—C9—H9A	120.0
N1—C1—N2	126.77 (14)	C9—C10—C11	120.61 (14)
N1—C1—N3	118.33 (15)	C9—C10—H10A	119.7
N2—C1—N3	114.87 (13)	C11—C10—H10A	119.7
N2—C2—H2A	109.5	C10—C11—C6	119.88 (14)
N2—C2—H2B	109.5	C10—C11—S1	124.60 (12)
H2A—C2—H2B	109.5	C6—C11—S1	115.51 (11)
N2—C2—H2C	109.5	C13—C12—S1	108.58 (10)
H2A—C2—H2C	109.5	C13—C12—H12A	110.0
H2B—C2—H2C	109.5	S1—C12—H12A	110.0
N2—C3—H3A	109.5	C13—C12—H12B	110.0
N2—C3—H3B	109.5	S1—C12—H12B	110.0
H3A—C3—H3B	109.5	H12A—C12—H12B	108.4
N2—C3—H3C	109.5	C18—C13—C14	118.55 (15)
H3A—C3—H3C	109.5	C18—C13—C12	121.19 (14)
H3B—C3—H3C	109.5	C14—C13—C12	120.25 (15)
N3—C4—H4A	109.5	C15—C14—C13	120.91 (16)
N3—C4—H4B	109.5	C15—C14—H14A	119.5
H4A—C4—H4B	109.5	C13—C14—H14A	119.5
N3—C4—H4C	109.5	C14—C15—C16	119.81 (16)
H4A—C4—H4C	109.5	C14—C15—H15A	120.1
H4B—C4—H4C	109.5	C16—C15—H15A	120.1
N3—C5—H5A	109.5	C15—C16—C17	120.10 (16)
N3—C5—H5B	109.5	C15—C16—H16A	120.0
H5A—C5—H5B	109.5	C17—C16—H16A	120.0
N3—C5—H5C	109.5	C16—C17—C18	119.97 (17)
H5A—C5—H5C	109.5	C16—C17—H17A	120.0
H5B—C5—H5C	109.5	C18—C17—H17A	120.0
C7—C6—N1	123.59 (13)	C13—C18—C17	120.66 (16)
C7—C6—C11	118.26 (13)	C13—C18—H18A	119.7
N1—C6—C11	117.77 (13)	C17—C18—H18A	119.7
C8—C7—C6	121.54 (14)		
			
C6—N1—C1—N2	28.2 (2)	C9—C10—C11—S1	−177.01 (12)
C6—N1—C1—N3	−153.64 (14)	C7—C6—C11—C10	−2.2 (2)
C3—N2—C1—N1	21.5 (2)	N1—C6—C11—C10	−175.37 (14)
C2—N2—C1—N1	−140.14 (17)	C7—C6—C11—S1	176.95 (12)
C3—N2—C1—N3	−156.70 (13)	N1—C6—C11—S1	3.76 (18)
C2—N2—C1—N3	41.6 (2)	C12—S1—C11—C10	−7.87 (16)
C4—N3—C1—N1	12.7 (2)	C12—S1—C11—C6	173.05 (12)
C5—N3—C1—N1	−135.83 (16)	C11—S1—C12—C13	−178.03 (11)
C4—N3—C1—N2	−168.95 (14)	S1—C12—C13—C18	−105.18 (15)
C5—N3—C1—N2	42.6 (2)	S1—C12—C13—C14	74.12 (16)
C1—N1—C6—C7	42.6 (2)	C18—C13—C14—C15	0.3 (2)
C1—N1—C6—C11	−144.63 (15)	C12—C13—C14—C15	−179.05 (14)
N1—C6—C7—C8	173.60 (15)	C13—C14—C15—C16	0.2 (2)
C11—C6—C7—C8	0.8 (2)	C14—C15—C16—C17	−0.8 (2)
C6—C7—C8—C9	0.7 (2)	C15—C16—C17—C18	0.9 (2)
C7—C8—C9—C10	−0.9 (2)	C14—C13—C18—C17	−0.2 (2)
C8—C9—C10—C11	−0.5 (2)	C12—C13—C18—C17	179.14 (14)
C9—C10—C11—C6	2.0 (2)	C16—C17—C18—C13	−0.4 (2)
